# Assessment of patient safety non-technical skills among consultants and residents in tertiary care hospitals: An analytical study

**DOI:** 10.12669/pjms.41.5.9914

**Published:** 2025-05

**Authors:** Ayesha Abubakar Mitha, Syed Fawad Mashhadi

**Affiliations:** 1 Ayesha Abubakar Mitha, MHPE. Training Branch, Armed Forces Post Graduate Medical Institute, Rawalpindi, Pakistan; 2 Syed Fawad Mashhadi, PhD. Department of Community Medicine & Public Health, Army Medical College, Rawalpindi, Pakistan

**Keywords:** Consultants, Lower and Middle Income countries, Non-technical skills, Patient safety, Residents

## Abstract

**Objective:**

To analyze Patient Safety Non-Technical Skills (PS NTSs) among consultants and residents for improving PS culture.

**Methods:**

A cross-sectional study was carried out among medical doctors (MBBS) working as Fellows of College of Physicians and Surgeons (FCPS) including residents and consultants in Medicine, Surgery and allied specialties in three tertiary care hospitals in Rawalpindi, Lahore and Sialkot from January to March 2023. Data was collected by face to face as well as using online Hospital Survey on PS Culture (HSOPSC) questionnaire. A total of 384 participants were enrolled in this study after taking informed consent. Average positive % responses with comparative responses among residents and consultants were calculated using appropriate statistical tests.

**Results:**

Positive response for Teamwork = 80.3%, System Based Practices = 61%, Communication within & inter units = 55.8% and 53.5% were the top three observations. Respondents with > five years of work experience were likely to practice PS 1.98 times > with one-five years (AOR = 1.98, 95% CI = 1.20 - 3.63, p value = 0.03). Working of respondents for 20 to 39 hours per week is protective towards PS (AOR=0.048, 95% CI = 0.006 - 0.390, p value = 0.04). Respondents working in specialty for one to five years are 5.44 times > other work durations to follow PS (AOR 5.447, 95% CI = 2.052 - 14.4, p value = 0.001).

**Conclusion:**

Poor adverse event reporting, lack of non-punitive responses by hospital administration and differences in PS NTS perceptions and practices among respondents were observed.

## INTRODUCTION

Patient Safety (PS) as an integral component of health care quality management affects countries at all levels of economic development.^1^ PS Non-Technical Skills (NTSs) are cognitive, social and personal resource skills complementing technical PS skills in contributing to safe and efficient clinical practices. Four effective PS NTS domains include Adverse Event Reporting, Teamwork, System Based Practices and Communication skills.^2^ Deficiencies in PS NTSs among doctors lead to serious medical accidents like wrong medication to patients or wrong side of surgery.^3^ Breakdowns in domains of PS NTSs remains a major root cause of harm inflicted to patients by healthcare workers.^4^ This situation is precarious in low and middle-income countries (LMICs) where insufficient infrastructures, suboptimal medical services and inadequate healthcare staff are challenges in providing quality healthcare.

Workplace training strategies lack development and implementation of PS NTSs among clinicians.^5^ Unsuccessful implementation of these skills are due to learner’s poor interest, lack of teaching faculty’s involvement and non- supportive hospital management.^6^ Organizations do not focus on effective adverse event reporting and feedback mechanisms due to which residents do not apply PS NTSs in their clinical practices.^7^ World Health Organization (WHO) has introduced PS NTSs curriculum for residents and for continuous medical education of consultants.^8^ PS curriculum has been introduced in medical institutes of some countries in their postgraduate training programs with limited studies on effectiveness of these training programs.^9^ Role of supervisors to train residents has been found deficient world over.^10^ In training hospitals of Pakistan, PS NTSs training is neither included in postgraduate training program curriculum nor in continuous professional development programs for clinicians.^11^,^12^

Exploring trends of PS NTSs among junior & senior clinicians would provide all stakeholders with evidence of existing PS culture.^13^ This study was aimed to assess the PS NTSs among consultants and residents for improving PS culture, to compare knowledge, attitude and practices among respondents as well as to identify roles of hospital management and of supervisors of residents in facilitating these practices, in order to provide a stimulus for medical educationists to formulate comprehensive PS curriculum in our settings.^14^

## METHODS

A cross sectional study was carried out among medical doctors (MBBS) working as Fellows of College of Physicians and Surgeons (FCPS) including residents and consultants in Medicine, Surgery and allied specialties in three tertiary care hospitals in Rawalpindi, Lahore and Sialkot from January to March 2023. A total of 384 participants including 192 residents and 192 consultants were assessed for Patient Safety Non-Technical Skills (PS NTSs). Sample size of 384 was calculated using the formula n = p (100–p) z²÷E² through probability stratified random sampling technique with consultants and residents as two strata, keeping the confidence interval at 95% and margin of error of 5%.

### Ethical approval:

It was taken from Ethical Review Committee (ERC) of Armed Forces Post Graduate Medical Institute vide Re.: 291-AAA-ERC-AFPGMI dated July 29, 2022.

### Inclusion criteria:

- Residents and consultants working in Medicine, Surgery and allied specialties belonging to both genders and ranging from age 25-50 years.

### Exclusion criteria:

- Consultants having only one year of clinical experience, first year residents and those who did not fill the questionnaire completely were excluded. During data collection every participant gave informed consent and was assured strict anonymity of questionnaire response.

The data was collected by face to face as well as using online Hospital Survey on PS Culture (HSOPSC) questionnaire.^15^ It includes four sub-scales for Teamwork, nine for System Based Practices, six for Communication, five for Adverse Event Reporting, six for Role of Hospital Management and four related to Role of Supervisors.^16^ A 5-point Likert scale is used to measure three to five items for each dimension in order to determine frequency (never to often) or agreement (strongly disagree to strongly agree). For the reported error questions, mistakes are defined as any type, without any regards to harm. The two single-item responses ask about the number of events recorded during the last 12 months and the overall PS score (excellent to failing). The responses “strongly disagree” and “disagree” were seen as unfavorable for PS culture, whereas the questions with a positive wording and replies like “agreed” and “strongly agreed” were considered good. A score of less than 50% indicated improvement in the dimension, whereas a score of more than 75% indicated development. The Agency for Healthcare Research and Quality (AHRQ) user guidelines were used to calculate the frequencies and positive response rate.

### Statistical analysis:

Data was analyzed using Statistical package for social sciences (SPSS) version 21. Frequencies and percentages of positive and negative responses for questionnaire items were calculated along with average positive percentage of each item as well as of main composite domain of those items. Chi-Square *x^2^* test was applied for comparing frequencies of responses of respondents. Binary Logistic Regression Model was applied for predicting associations between categorical outcome variables with their background variables. Chi- Square *x^2^* and Hosmer Lemeshow tests were initially conducted to establish Goodness of Fit for logistic regression. A p-value of <0.05 was taken as significant.

## RESULTS

**Fig.1:**
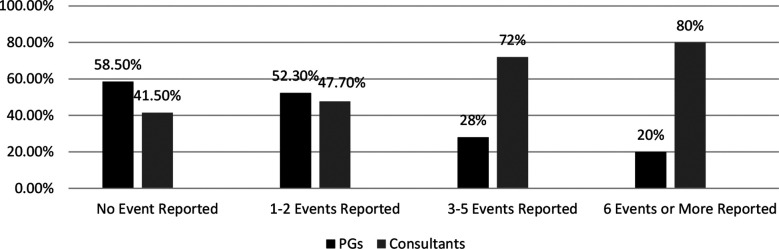
Comparative Adverse Event Reporting of Respondents-Residents referred to as Postgraduate Trainees (PGs).

**Fig.2:**
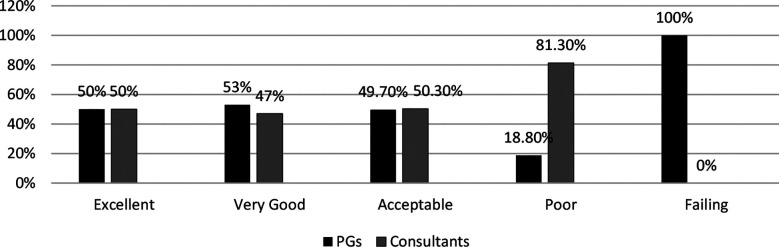
Comparative PS Grading of Hospitals by Respondents - Residents referred to as Postgraduate Trainees (PGs).

The age of 384 participants ranged between 25-50 years with mean age of 35.7 years. The association of socio-demographic details with different variables is shown in Table-I. Average Positive Percentage Responses reflect highest response (80.3%) for Teamwork and in descending order for System Based Practices (61%), Communication within & inter units (55.8% & 53.5%) and lowest response for Adverse Event Reporting skill was (38%). There was a facilitating role of supervisors (76.7%) and of hospital administration (61%) in PS NTS practices by clinicians. However, feedback on reported adverse events by hospital administrations was 49.5% with non-punitive response to reported errors as only 35.5%. Respondents with > five years of work experience in current workplace are predicted to practice PS measures 1.98 times more than respondents with one to five years of experience (AOR = 1.98, 95% CI= 1.20 - 3.63, p= 0.03). Results also predict that working of respondents for 20 to 39 hours per week is protective towards PS practices (AOR=0.048, 95% CI = 0.006 - 0.390, p= 0.04). Respondents with a work experience of one to five years are predicted to 5.44 times more likely to follow PS practices as compared to the rest of the groups (AOR 5.447, 95% CI = 2.052 - 14.4, p = 0.001). These results are depicted in Table II. Overall 45.8% of participants had not reported any adverse event including 58.5% residents and 41.5% consultants. About 33.3% respondents had reported only one to two events, 19.5% had reported three to five and only 1.3% had reported six or more adverse events. The comparative response of residents and consultants was assessed, as shown in Fig.1. About 43.8% of participants graded their hospital as very good, 43.5% graded it as acceptable, 8.3% considered it as excellent and only 4.2% as Poor. A comparative response of residents and consultants is shown in Fig.2.

**Table-I:** Socio-demographic details of respondents.

Variable	Categories	Frequency	Percentage	PGs (%)	Consultants (%)	p - value
Category of Respondents	Resident (PG)	192	50.0	-		
	Consultant	192	50.0			
Year of residency program of residents	2nd Year	101	26.3	-		
	3rd Year	53	13.8			
	4th Year	33	8.6			
	5th Year	5	1.3			
Gender	Male	262	68.2	124(47.3)	138(52.7)	0.125
	Female	122	31.8	68(55.7)	54(44.3)	
Tertiary care Teaching hospitals	Lahore	162	42.2	88(54.3)	74(45.7)	0.001
	Rawalpindi	131	34.1	34(26.0)	97(74.0)	
	Sialkot	91	23.7	70(76.9)	21(23.1)	
Primary working area	Gen Medicine	56	14.6	36(64.3)	20(35.7)	0.002
	Gen Surgery	52	13.5	27(51.9)	25(48.1)	
	Obs /Gynecology	47	12.2	29(61.7)	18(38.3)	
	Pediatrics	45	11.7	22(48.9)	23(51.1)	
	Emergency	2	0.5	0(0.0)	2(100.0)	
	ICU	4	1.0	0(0.0)	4(100.0)	
	Psychiatry	1	0.3	0(0.0)	1(100.0)	
	Dermatology	17	4.4	5(29.4)	12(70.6)	
	Nephrology	3	0.8	2(66.7)	1(33.3)	
	Ortho Surgery	12	3.1	2(16.7)	10(83.3)	
	Radiology	42	10.9	34(81.0)	8(19.0)	
	Anesthesiology	35	9.1	18(51.4)	17(48.6)	
	Other specialty	68	17.7	17(20)	51(75.0)
*Background Information*						
Duration of Job	1 to 5 years	358	93.2	187(52.2)	171(47.8)	0.001
	> 5 years	26	6.8	5(19.2)	21(80.8)	
Work hours per week	< 20 hours	2	0.5	1(50.0)	1(50.0)	0.932
	20 to 39 hours	38	9.9	20(52.6)	18(47.4)	
	> 40 hours	344	89.6	171(49.7)	173(50.3)	
Duration of working in specialty	< 1 year	31	8.1	28(90.3)	3(9.7)	0.003
	1 to 5 years	196	51.0	159(81.1)	37(18.9)	
	6 to 10 years	41	10.7	1(2.4)	40(97.6)	
	11 to 15 years	67	17.4	2(3.0)	65(97.0)	
	> 15 years	49	12.8	2(4.1)	47(95.9)	

**Table-II:** Binary logistic regression analysis - PS practices in hospitals with covariates / background variables

Independent variable	Category	**UOR	95% CI		p-value	***AOR	95% CI		p-value
		Lower	Upper	Lower	Upper
Duration of Job	Reference value: 1 to 5 years								
	> 5 yrs.	2.15	1.33	3.45	0.014	1.98	1.20	3.63	0.03
Work hours per week	Reference value: < 20 hours per week								
	20 to 39 hours/wk	0.046	0.006	0.360	0.003	0.048	0.006	0.390	0.04
	>40 hours/ wk	0.388	0.051	2.979	0.388	0.389	0.049	3.066	0.37
Duration of working in specialty	Reference value: < 1 year								
	1 to 5 yrs.	7.605	3.187	18.15	0.001	5.447	2.052	14.4	0.001
	6 to 10 yrs.	28.89	3.507	23.79	0.002	20.79	2.346	18.42	0.116
	11 to 15 yrs.	3.677	1.404	9.62	0.001	2.424	0.81	7.24	0.113
	> 15 yrs.	3.701	1.308	10.47	0.014	1.899	0.58	6.15	0.285

*wk= weeks, yrs = years,

** UOR = Unadjusted Odds Ratio,

*** AOR = Adjusted Odds Ratio

## DISCUSSION

A positive response for Teamwork, System Based Practices, Communication within & inter units were the top three observations from this study. Results were based on domains of PS NTSs prevalent and lacking in practices of consultants and residents. It was also assessed how supervisors were promoting PS practices in their residents and whether hospital management was supportive of PS culture or not. The number of adverse events reported were measured as well as how respondents graded their hospitals on PS practices.

In Pakistan, one of the earlier PS studies limited to only department of General Medicine of a local hospital was conducted in 2018 with mostly similar results except System Based Practices being top ranked.^17^ HSOPSC was found effective for collecting data on concepts, opinions , attitudes and practices of PS in HICs as well as in LMICs.^18^

A systematic review followed by meta-analysis on Teamwork skill among Asian countries has identified 82% prevalence of Teamwork skill among hospital staff including senior and junior doctors.^18^ Similarly, a survey conducted by Agency of Healthcare Research & Quality (AHRQ) USA in 2021 on practices of teamwork within units found a prevalence of 82% in USA and 73.5% in Sweden.^19^,^20^ Healthcare workers have been experiencing good peer support for improved patient care. However, a study in Pakistan on Teamwork skill ranked it at 4^th^ position while in another study it was 81.7% in weightage.^17^,^21^ The results of this study are similar to some international studies as 80.3% participants gave the highest average positive response on Teamwork within their units and that the teamwork skills are strongly evident in residents as compared to consultants.

Clinical practices based on well-defined, system based practices of the hospitals encompass the procedures and ways of performances focusing towards preventing medical errors by staff.^22^ In AHRQ 2021 survey, average positive response on System Based Practices was 71%, in Shenzhen hospitals it was 87.2% and in Sweden it was 61.5%.^19^,^23^ In one study in Pakistan, system based practices including situational awareness skill was ranked as a top skill in clinical practices and in another study it was given 86.5% weightage.^17^,^21^ In this study, respondents demonstrated good knowledge of efficiency level of health care practices in hospitals and established strategies for PS enhancement in routine and in crises situations being parts of system based practices with 61% as an average positive response to these practices. It was acknowledged by 80%, which showed that they were actively taking measures to improve PS and 65% had evaluated the effectiveness of steps taken for PS in their hospitals. While 79% believed in improvement of hospital systems through reported mistakes. Only 28% believed that they mostly worked in a crisis mode that was adversely affecting PS. These results reflect that residents responded more (52.4%) as compared to consultants (47.6%) on actively doing things to improve PS (p= 0.05). A clear majority of residents (75%) strongly agreed that PS was never sacrificed for more work done (p= 0.002). PS problems need to be improved with more diligent system based clinical practices.

In this study, while analyzing the communication within units and inter units the average positive response on communication within units was low. Around half of respondents acknowledged that staff could openly communicate with seniors regarding issues related to PS and only few believed that staff could challenge the opinions of senior staff. Majority of consultants agreed that staff can freely speak against PS issues than residents (p= 0.002). However, staff do not feel free to question their seniors being a lapse in communication as most of the consultants think it to be occurring rarely as compared to only some residents (p= 0.003). The communication barrier with senior staff members is a default norm which needs to be addressed if PS perspective is to be kept as one of the main focus of patient management. Hospital units were found to coordinate well but PS related issues exist in across hospital unit communication. In a study conducted in Sweden, the positive response was 66.3%.^20^ In Asian countries, it was 66% and in Shenzhen states, it was 67.2%.^18^,^22^ Communication openness was ranked 3^rd^ in a study in Pakistan.^17^ In another Pakistani study it was 65.4%.^21^

In Sweden positive response of adverse event reporting was 54.4%.^20^ The non-punitive response to error reporting by administration was 67.2% and the feedback from higher authorities was 64.8%.^20^ In Asian countries, it was 67% positive response with non-punitive response of only 47% and feedback given as 69%.^18^ In Shenzhen states, it was a good response of 72.2% and an impressive feedback response by administration of 86.7%.^22^ Average positive response in AHRQ 2021 Survey in USA was 68% with non- punitive response of 49% and feedback given was 69%.^19^ In a Pakistani study it was a relatively poor response of 53.7% with non-punitive response of 32.1% and a good response on feedback by administration of 73.7%.^21^ In this study the average positive response on frequency of adverse event reporting was very low (38%) with non-punitive response to error reporting being 35.5% and feedback from administration is 49.5%. Chi-square shows that out of 109 respondents, more consultants (60.6%) as compared to residents (39.4%) believed that most of the time staff thought that their mistakes were kept in their personal files. In HICs like North America, one quarter of medical schools taught PS with limited hours while in LMICs majority of healthcare institutes are at a planning stage of implementing PS culture.^23^ Punitive responses to medical errors also form a barrier for staff to report PS related mistakes.^24^

Poor involvement of organizational administration and generally an unsupportive institutional culture towards the concepts of PS hinders the implementation process.^25^ In Sweden the supportive role of hospital management was appreciated by 47.9% of respondents.^20^ In a study among Asian countries, the facilitating role was 72%.^18^ In Shenzhen states it was a good average response of 84.9%.^22^ In AHRQ 2021 Survey it was 69%.^20^ In Pakistan it was a relatively good response of 71.6%.^22^ A majority of respondents (77%) believe that hospital management is providing a PS promoting working climate. Total 63% respondents have the perception that PS is a top concern of hospital management but more prioritized attention is still required as 43% respondents believed that hospital management only supports PS practices after happening of an adverse event. Differences in perceptions of respondents affects the overall quality of patient care.

In Turkey, the positive perception for supervisor’s facilitating role was 52.1%.^18^ In Sweden, it was appreciated by 66.7% of respondents.^20^ In Asian countries, it was a good average positive response of 80%.^18^ AHRQ 2021 survey reflected a 80% response and in Shenzhen states, it was 78.2%.^19^,^22^ In another Pakistani study, it was a relatively average response of 65.8%.^21^ In this study, the average positive response was 76.7%. Out of 192 respondents, 91% residents agreed that their supervisors appreciated their PS practices in hospitals and positively considered staff suggestions on improving PS. However, only 23% residents expressed that they had to rush with patient management due to excessive workloads.

### Limitations:

Comprehensive quantitative studies on PS NTS practices among Residents and Consultants in Pakistan are not available with limited requisite statistical data for comparison. Significant assessment of PS culture of an organization requires serial comparative similar studies in the same hospitals with trends of PS culture over time, which were also not available.

## CONCLUSION

Poor adverse event reporting, lack of non-punitive responses by hospital administration and differences in PS NTS perceptions and practices among respondents were observed in the study.

### Recommendations:

PS measures are being sub optimally practiced even in teaching hospitals of Pakistan. Poor Adverse Event Reporting, lack of non-punitive responses by hospital administration and differences in PS NTS perceptions among respondents can improve from stakeholders’ collaborative approach towards postgraduate PS NTS training curricula development. Survey can be utilized as a benchmark for enhancing current PS practices and for future PS comparative reevaluations of these hospitals through HSOPSC -1. For collaborative efforts to be executed by managers of all clinical departments and hospital administrators, PS workshops by PS experts as well as comprehensive and practical postgraduate PS curricula development and rigorous training programs be conducted. Administrations need to focus on non-punitive response approach towards reporting of medical errors and provision of prompt constructive feedback. Clinical auditors as well as governing bodies of teaching hospitals should contribute at appropriate levels to synchronize efforts towards enhancing composite PS measures.

### Authors’ Contribution:

**AAM:** Conceived, designed, did data collection, statistical analysis, manuscript writing and responsible for integrity of research.

**SFM** Guided in designing, did editing of manuscript, reviewed & final approval of manuscript.

## References

[1] Dimitriadou M, Merkouris A, Charalambous A, Lemonidou C, Papastavrou E (2021). The knowledge about patient safety among undergraduate nurse students in Cyprus and Greece:a comparative study. BMC Nurs.

[2] Gordon M, Darbyshire D, Baker P (2012). Non-technical skills training to enhance patient safety:A systematic review. Med Educ.

[3] Uramatsu M, Fujisawa Y, Mizuno S, Souma T, Komatsubara A, Miki T (2017). Do failures in non-technical skills contribute to fatal medical accidents in Japan?A review of the 2010-2013 national accident reports. BMJ Open.

[4] Gillespie BM, Harbeck EL, Lavin J, Hamilton K, Gardiner T, Withers TK (2018). Evaluation of a patient safety programme on surgical safety checklist compliance:A prospective longitudinal study. BMJ Open Qual.

[5] Bari A, Jabeen U, Bano I, Rathore AW (2017). Patient safety awareness among postgraduate students and nurses in a tertiary health care facility. Pak J Med Sci.

[6] Ounounou E, Aydin A, Brunckhorst O, Khan MS, Dasgupta P, Ahmed K (2019). Nontechnical Skills in Surgery:A Systematic Review of Current Training Modalities. J Surg Educ.

[7] Garcia CDL, De Abreu LC, Ramos JLS, De Castro CFD, Smiderle FRN, Dos Santos JA (2019). Influence of burnout on patient safety:systematic review and meta-analysis. Med.

[8] Sujan MA, Furniss D, Anderson J, Braithwaite J, Hollnagel E (2019). Resilient Health Care as the basis for teaching patient safety –A Safety-II critique of the World Health Organisation patient safety curriculum. Saf Sci.

[9] Giroldi E, Veldhuijzen W, Geelen K, Muris J, Bareman F, Bueving H (2017). Developing skilled doctor –patient communication in the workplace:a qualitative study of the experiences of trainees and clinical supervisors. Adv Heal Sci Educ.

[10] Mundschenk MB, Odom EB, Ghosh TD, Kleiber GM, Yee A, Patel KB (2018). Are Residents Prepared for Surgical Cases?Implications in Patient Safety and Education. J Surg Educ.

[11] Jalil A, Mahmood QK, Fischer F (2020). Young medical doctors'perspectives on professionalism:A qualitative study conducted in public hospitals in Pakistan. BMC Health Serv Res.

[12] Shah M, Perveen S (2016). State of Healthcare Quality and Patient Safety in Pakistan. Pak J Public Heal.

[13] Al Salem G, Bowie P, Morrison J (2019). Hospital Survey on Patient Safety Culture:Psychometric evaluation in Kuwaiti public healthcare settings. BMJ Open.

[14] Tartaglia Reis C, Guerra Paiva S, Sousa P (2018). The patient safety culture:a systematic review by characteristics of Hospital Survey on Patient Safety Culture dimensions. Int J Qual Heal Care.

[15] Waterson P, Carman EM, Manser T, Hammer A (2019). Hospital Survey on Patient Safety Culture (HSOPSC):A systematic review of the psychometric properties of 62 international studies. BMJ Open.

[16] Palmieri PA, Leyva-Moral JM, Camacho-Rodriguez DE, Granel-Gimenez N, Ford EW, Mathieson KM (2020). Hospital survey on patient safety culture (HSOPSC):A multi-method approach for target-language instrument translation, adaptation, and validation to improve the equivalence of meaning for cross-cultural research. BMC Nurs.

[17] Majeed N, Mehboob U (2018). Doctors'experiences and awareness of non-technical skills, a way to the development of a behavioral marker system for patient management. Health Prof Educ J.

[18] Damayanti RA, Bachtiar A (2019). Outcome of Patient Safety Culture Using The Hospital Survey on Patient Safety Culture (HSOPSC) In Asia:A Systematic Review With Meta Analysis. Proc Int Conf Appl Sci Heal.

[19] (2022). HSOPSC Version 1.0:2021 User Database Report AHRQ.

[20] Danielsson M, Nilsen P, Rutberg H, Arestedt K (2019). A National Study of Patient Safety Culture in Hospitals in Sweden. J Patient Saf.

[21] Hameed S, Sheikh AH, Yaqoob M, Latif MZ (2021). Patient safety culture:a survey of private sector tertiary care hospital of Lahore, Pakistan. Pak Biomed J.

[22] Hao HS, Gao H, Li T, Zhang D (2020). Assessment and comparison of patient safety culture among health-care providers in shenzhen hospitals. Risk Manag Healthc Policy.

[23] Tussardi IT, Benoni R, Moretti F, Tardivo S, Poli A, Wu AW (2021). Patient safety in the eyes of aspiring healthcare professionals:A systematic review of their attitudes. Int J Environ Res Public Health.

[24] Ginsburg LR, Dhingra-Kumar N, Donaldson LJ (2017). What stage are low-income and middle-income countries (LMICs) at with patient safety curriculum implementation and what are the barriers to implementation?A two-stage cross-sectional study. BMJ Open.

[25] Lin Y, Scott JW, Yi S, Taylor KK, Ntakiyiruta G, Ntirenganya F (2018). Improving Surgical Safety and Nontechnical Skills in Variable-Resource Contexts:A Novel Educational Curriculum. J Surg Educ.

